# *trans*-Dichloridobis[tris­(4-methoxy­phen­yl)phosphine]palladium(II) benzene monosolvate

**DOI:** 10.1107/S1600536809046261

**Published:** 2009-11-07

**Authors:** Charmaine van Blerk, Cedric W. Holzapfel

**Affiliations:** a University of Johannesburg, Department of Chemistry, PO Box 524, Auckland Park, Johannesburg 2006, South Africa

## Abstract

The structure of the title compound, [PdCl_2_(C_21_H_21_O_3_P)_2_]·C_6_H_6_, shows a square-planar geometry for the Pd^II^ atom within a Cl_2_[P(PhOMe)_3_]_2_ ligand set. The crystal structure contains benzene as solvent. The Pd^II^ atom sits on a centre of inversion and therefore the asymmetric unit contains the Pd^II^ atom, one Cl atom, one tris­(4-methoxy­phen­yl)phosphine ligand and one half of the benzene solvent mol­ecule.

## Related literature

For related structures and literature on similar palladium complexes, see: Robertson & Cole-Hamilton (2002 ▶); Van Leeuwen *et al.* (2003 ▶); Williams *et al.* (2008 ▶).
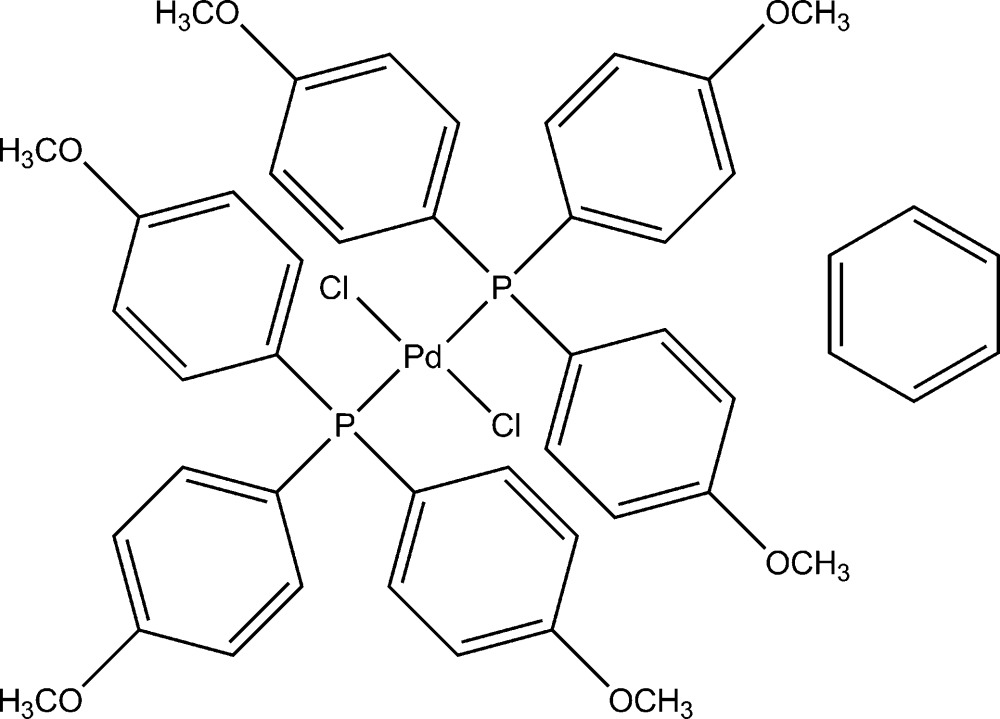


## Experimental

### 

#### Crystal data

[PdCl_2_(C_21_H_21_O_3_P)_2_]·C_6_H_6_
*M* *_r_* = 960.10
Triclinic, 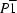

*a* = 7.9338 (2) Å
*b* = 12.1886 (3) Å
*c* = 12.5268 (3) Å
α = 85.981 (3)°
β = 78.840 (2)°
γ = 76.155 (2)°
*V* = 1153.57 (5) Å^3^
*Z* = 1
Mo *K*α radiation
μ = 0.63 mm^−1^
*T* = 295 K
0.34 × 0.24 × 0.10 mm

#### Data collection

Bruker SMART CCD diffractometer
Absorption correction: multi-scan (**APEX2* AX-Scale*; Bruker, 2008 ▶) *T*_min_ = 0.813, *T*_max_ = 0.939
31847 measured reflections
5781 independent reflections
4546 reflections with *I* > 2σ(*I*)
*R*_int_ = 0.046

#### Refinement

*R*[*F* ^2^ > 2σ(*F* ^2^)] = 0.038
*wR*(*F* ^2^) = 0.097
*S* = 1.08
5781 reflections
271 parameters
H-atom parameters constrained
Δρ_max_ = 1.26 e Å^−3^
Δρ_min_ = −0.53 e Å^−3^

### 

Data collection: *SMART-NT* (Bruker, 1999 ▶); cell refinement: *SAINT* (Bruker, 2008 ▶); data reduction: *SAINT*; program(s) used to solve structure: *SHELXS97* (Sheldrick, 2008 ▶); program(s) used to refine structure: *SHELXL97* (Sheldrick, 2008 ▶); molecular graphics: *X-SEED* (Barbour, 2001 ▶) and *Mercury* (Macrae *et al.*, 2006 ▶); software used to prepare material for publication: *publCIF* (Westrip, 2009 ▶).

## Supplementary Material

Crystal structure: contains datablocks I, global. DOI: 10.1107/S1600536809046261/ez2192sup1.cif

Structure factors: contains datablocks I. DOI: 10.1107/S1600536809046261/ez2192Isup2.hkl

Additional supplementary materials:  crystallographic information; 3D view; checkCIF report

## Figures and Tables

**Fig. 1.**
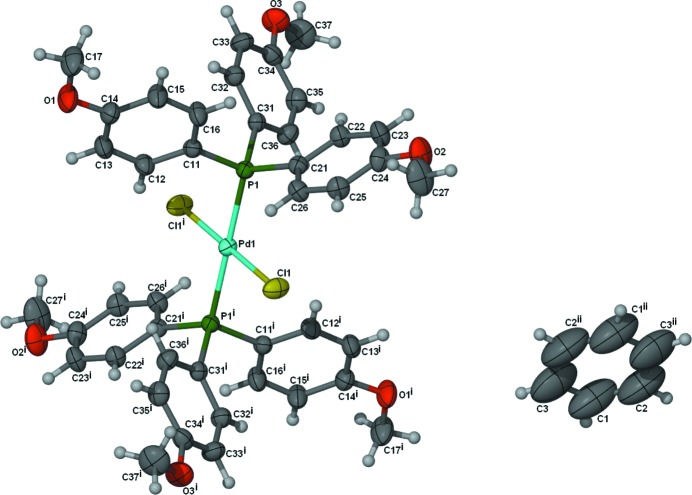
: Molecular structure of (I) with displacement ellipsoids drawn at the 50% probability level. Atoms labelled with (i) are at symmetry position (– x, – y, – z) and atoms labelled with (ii) are at symmetry position (– x + 1, – y + 1, – z + 1)

## supplementary crystallographic information

### Comment

The palladium-catalysed methoxycarbonylation (Robertson & Cole-Hamilton, 2002) of 1-alkenes is an active area of research. The palladium complexes (Ar_3_P)_2_Pd*X*_2_ (*X* = Cl, DMS, OTf *etc*.) are the preferred catalysts but most quantitative studies have been carried out with complexes where the phosphine ligand is limited to triphenylphosphine. The X-ray structures (Van Leeuwen *et al.*, 2003 and Williams *et al.*, 2008) of several of this class of palladium(II) complexes have been determined. Our studies (Williams *et al.*, 2008) on the effect of substituents on the triarylphosphine ligands on regioselectivity and reaction rate showed *trans*-dichloro-bis[tris-(4-methoxy)phosphine] palladium(II) to be an exceptionally efficient catalyst for the production of linear esters from 1-alkenes at high rates.

The structure of the title compound (I), [PdCl_2_(C_42_H_42_P_2_O_6_).C_6_H_6_] shows a square planar geometry for the Pd^II^ atom within the Cl_2_(P(PhOMe)_3_) ligand set. The crystal structure contains benzene as a solvate. The solvent molecule exhibits noticeable disorder but this disorder was not modelled. The palladium atom sits on a centre of inversion and therefore the asymmetric unit contains the palladium atom, one chlorine atom, one tris-(4-methoxyphenyl)phenylphosphine ligand and one half of the benzene solvent molecule.

### Experimental

Tris-(4-methoxyphenyl)phosphine (704 mg, 0.2 mmol) was added to a solution of lithium chloride (85 mg, 0.2 mmol) and palladium(II) chloride (177 mg, 0.1 mmol) in 15 ml me thanol. The mixture was heated under reflux in an atmosphere of nitrogen for 1 h resulting in the formation of the product as a yellow precipitate. The solution was allowed to cool to room temperature and the product (710 mg) was collected by filtration, washed with fresh methanol and dried under vacuum. The product was recrystallized from 1:1 ethyl acetate:benzene to furnish yellow plates (m. p. > 250°C, decomp.) A suitable single-crystal was selected for the single-crystal X-ray diffraction analysis.

### Refinement

H atoms were geometrically positioned and refined in the riding-model approximation, with C—H = 0.97 Å, N—H = 0.89 Å, and *U*_iso_(H) = 1.2Ueq(C) or 1.5Ueq(N). For (I), the highest peak in the final difference map is 0.98Å from C15 and the deepest hole is 0.01Å from Pd1.

### Crystal data

**Table:**

[PdCl_2_(C_21_H_21_O_3_P)_2_]·C_6_H_6_	*Z* = 1
*M**_r_* = 960.10	*F*(000) = 494
Triclinic, *P*1	*D*_x_ = 1.382 Mg m^−^^3^
Hall symbol: -P 1	Mo *K*α radiation, λ = 0.71073 Å
*a* = 7.9338 (2) Å	Cell parameters from 9954 reflections
*b* = 12.1886 (3) Å	θ = 1.7–28.3°
*c* = 12.5268 (3) Å	µ = 0.63 mm^−^^1^
α = 85.981 (3)°	*T* = 295 K
β = 78.840 (2)°	Flat, yellow
γ = 76.155 (2)°	0.34 × 0.24 × 0.10 mm
*V* = 1153.57 (5) Å^3^	

### Data collection

**Table:**

Bruker SMART CCD diffractometer	5781 independent reflections
Radiation source: fine-focus sealed tube	4546 reflections with *I* > 2σ(*I*)
graphite	*R*_int_ = 0.046
φ and ω scans	θ_max_ = 28.4°, θ_min_ = 1.7°
Absorption correction: multi-scan (*APEX2 Ax-Scale*; Bruker, 2008)	*h* = −10→10
*T*_min_ = 0.813, *T*_max_ = 0.939	*k* = −16→16
31847 measured reflections	*l* = −16→16

### Refinement

**Table:**

Refinement on *F*^2^	Primary atom site location: structure-invariant direct methods
Least-squares matrix: full	Secondary atom site location: difference Fourier map
*R*[*F*^2^ > 2σ(*F*^2^)] = 0.038	Hydrogen site location: inferred from neighbouring sites
*wR*(*F*^2^) = 0.097	H-atom parameters constrained
*S* = 1.08	*w* = 1/[σ^2^(*F*_o_^2^) + (0.04*P*)^2^ + 0.8985*P*] where *P* = (*F*_o_^2^ + 2*F*_c_^2^)/3
5781 reflections	(Δ/σ)_max_ < 0.001
271 parameters	Δρ_max_ = 1.26 e Å^−^^3^
0 restraints	Δρ_min_ = −0.53 e Å^−^^3^

### Special details

**Table:**

Geometry. All e.s.d.'s (except the e.s.d. in the dihedral angle between two l.s. planes) are estimated using the full covariance matrix. The cell e.s.d.'s are taken into account individually in the estimation of e.s.d.'s in distances, angles and torsion angles; correlations between e.s.d.'s in cell parameters are only used when they are defined by crystal symmetry. An approximate (isotropic) treatment of cell e.s.d.'s is used for estimating e.s.d.'s involving l.s. planes.
Refinement. Refinement of *F*^2^ against ALL reflections. The weighted *R*-factor *wR* and goodness of fit *S* are based on *F*^2^, conventional *R*-factors *R* are based on *F*, with *F* set to zero for negative *F*^2^. The threshold expression of *F*^2^ > σ(*F*^2^) is used only for calculating *R*-factors(gt) *etc*. and is not relevant to the choice of reflections for refinement. *R*-factors based on *F*^2^ are statistically about twice as large as those based on *F*, and *R*- factors based on ALL data will be even larger.

### Fractional atomic coordinates and isotropic or equivalent isotropic displacement parameters (Å^2^)

**Table:**

	*x*	*y*	*z*	*U*_iso_*/*U*_eq_	
C1	0.4425 (16)	0.4281 (7)	0.5761 (6)	0.155 (3)	
H1	0.4046	0.3780	0.6295	0.186*	
C2	0.5827 (15)	0.4708 (8)	0.5889 (6)	0.157 (3)	
H2	0.6361	0.4503	0.6495	0.188*	
C3	0.3558 (14)	0.4537 (7)	0.4915 (8)	0.164 (3)	
H3	0.2608	0.4232	0.4866	0.197*	
C11	0.0548 (3)	0.1003 (2)	−0.2660 (2)	0.0322 (5)	
C12	−0.0842 (4)	0.0505 (3)	−0.2715 (2)	0.0394 (6)	
H12	−0.1180	0.0008	−0.2162	0.047*	
C13	−0.1725 (4)	0.0744 (3)	−0.3587 (2)	0.0457 (7)	
H13	−0.2650	0.0406	−0.3614	0.055*	
C14	−0.1248 (4)	0.1475 (3)	−0.4413 (2)	0.0415 (6)	
C15	0.0146 (4)	0.1971 (3)	−0.4377 (2)	0.0491 (8)	
H15	0.0490	0.2459	−0.4937	0.059*	
C16	0.1017 (4)	0.1736 (3)	−0.3504 (2)	0.0451 (7)	
H16	0.1941	0.2076	−0.3481	0.054*	
C17	−0.1700 (5)	0.2373 (4)	−0.6136 (3)	0.0663 (10)	
H17A	−0.1778	0.3112	−0.5882	0.099*	
H17B	−0.2476	0.2429	−0.6647	0.099*	
H17C	−0.0508	0.2058	−0.6484	0.099*	
C21	0.2612 (3)	0.1876 (2)	−0.1422 (2)	0.0319 (5)	
C22	0.4426 (4)	0.1803 (2)	−0.1504 (2)	0.0379 (6)	
H22	0.5220	0.1107	−0.1627	0.046*	
C23	0.5046 (4)	0.2750 (3)	−0.1406 (3)	0.0462 (7)	
H23	0.6252	0.2685	−0.1460	0.055*	
C24	0.3887 (4)	0.3800 (2)	−0.1227 (2)	0.0419 (7)	
C25	0.2087 (4)	0.3885 (2)	−0.1136 (2)	0.0417 (7)	
H25	0.1297	0.4582	−0.1011	0.050*	
C26	0.1472 (4)	0.2935 (2)	−0.1233 (2)	0.0383 (6)	
H26	0.0264	0.3004	−0.1169	0.046*	
C27	0.3489 (6)	0.5791 (3)	−0.1038 (4)	0.0781 (13)	
H27A	0.2900	0.5975	−0.1650	0.117*	
H27B	0.4168	0.6336	−0.0997	0.117*	
H27C	0.2628	0.5801	−0.0382	0.117*	
C31	0.3704 (3)	−0.0450 (2)	−0.2019 (2)	0.0305 (5)	
C32	0.4241 (4)	−0.0750 (2)	−0.3103 (2)	0.0381 (6)	
H32	0.3637	−0.0348	−0.3627	0.046*	
C33	0.5670 (4)	−0.1644 (3)	−0.3409 (2)	0.0465 (7)	
H33	0.6017	−0.1840	−0.4136	0.056*	
C34	0.6591 (4)	−0.2253 (2)	−0.2634 (3)	0.0408 (6)	
C35	0.6116 (4)	−0.1934 (3)	−0.1561 (3)	0.0438 (7)	
H35	0.6766	−0.2308	−0.1046	0.053*	
C36	0.4660 (4)	−0.1053 (2)	−0.1256 (2)	0.0406 (6)	
H36	0.4315	−0.0861	−0.0528	0.049*	
C37	0.8756 (5)	−0.3895 (3)	−0.2234 (4)	0.0722 (11)	
H37A	0.9291	−0.3483	−0.1822	0.108*	
H37B	0.9644	−0.4495	−0.2604	0.108*	
H37C	0.7882	−0.4206	−0.1751	0.108*	
O1	−0.2204 (3)	0.1654 (2)	−0.52266 (18)	0.0592 (6)	
O2	0.4641 (3)	0.46844 (19)	−0.1165 (2)	0.0626 (7)	
O3	0.7933 (3)	−0.31468 (19)	−0.3017 (2)	0.0576 (6)	
P1	0.17506 (8)	0.06402 (5)	−0.15376 (5)	0.02933 (15)	
Cl1	−0.05138 (11)	0.16975 (6)	0.08394 (6)	0.04878 (19)	
Pd1	0.0000	0.0000	0.0000	0.02850 (9)	

### Atomic displacement parameters (Å^2^)

**Table:**

	*U*^11^	*U*^22^	*U*^33^	*U*^12^	*U*^13^	*U*^23^
C1	0.264 (12)	0.110 (5)	0.087 (5)	−0.034 (6)	−0.030 (6)	−0.009 (4)
C2	0.244 (11)	0.137 (7)	0.080 (5)	−0.018 (7)	−0.037 (6)	−0.015 (5)
C3	0.256 (11)	0.129 (6)	0.109 (6)	−0.046 (7)	−0.024 (7)	−0.038 (5)
C11	0.0305 (13)	0.0349 (14)	0.0304 (13)	−0.0055 (11)	−0.0069 (10)	0.0006 (10)
C12	0.0387 (15)	0.0453 (16)	0.0373 (14)	−0.0170 (13)	−0.0083 (12)	0.0075 (12)
C13	0.0408 (16)	0.0573 (19)	0.0461 (16)	−0.0236 (14)	−0.0117 (13)	0.0031 (14)
C14	0.0414 (15)	0.0484 (17)	0.0375 (15)	−0.0109 (13)	−0.0138 (12)	0.0001 (13)
C15	0.0554 (19)	0.066 (2)	0.0372 (15)	−0.0340 (16)	−0.0158 (14)	0.0154 (14)
C16	0.0459 (16)	0.0534 (18)	0.0454 (16)	−0.0264 (14)	−0.0173 (13)	0.0123 (14)
C17	0.078 (3)	0.084 (3)	0.051 (2)	−0.036 (2)	−0.0331 (19)	0.0202 (19)
C21	0.0367 (14)	0.0303 (13)	0.0295 (12)	−0.0101 (11)	−0.0070 (10)	0.0036 (10)
C22	0.0368 (14)	0.0323 (14)	0.0446 (15)	−0.0063 (11)	−0.0088 (12)	−0.0016 (12)
C23	0.0355 (15)	0.0440 (17)	0.063 (2)	−0.0147 (13)	−0.0126 (14)	0.0005 (14)
C24	0.0517 (17)	0.0324 (15)	0.0478 (16)	−0.0156 (13)	−0.0181 (14)	0.0039 (12)
C25	0.0450 (16)	0.0287 (14)	0.0496 (16)	−0.0043 (12)	−0.0109 (13)	0.0018 (12)
C26	0.0340 (14)	0.0343 (14)	0.0464 (16)	−0.0079 (11)	−0.0089 (12)	0.0042 (12)
C27	0.079 (3)	0.0346 (18)	0.129 (4)	−0.0176 (18)	−0.033 (3)	0.000 (2)
C31	0.0321 (13)	0.0273 (13)	0.0333 (13)	−0.0080 (10)	−0.0079 (10)	0.0000 (10)
C32	0.0359 (14)	0.0416 (16)	0.0358 (14)	−0.0051 (12)	−0.0091 (11)	−0.0008 (12)
C33	0.0428 (16)	0.0565 (19)	0.0360 (15)	−0.0031 (14)	−0.0041 (12)	−0.0101 (13)
C34	0.0316 (14)	0.0369 (15)	0.0525 (17)	−0.0071 (12)	−0.0040 (12)	−0.0053 (13)
C35	0.0431 (16)	0.0401 (16)	0.0491 (17)	−0.0053 (13)	−0.0182 (13)	0.0048 (13)
C36	0.0462 (16)	0.0403 (16)	0.0345 (14)	−0.0048 (13)	−0.0124 (12)	−0.0008 (12)
C37	0.061 (2)	0.054 (2)	0.090 (3)	0.0119 (18)	−0.018 (2)	−0.001 (2)
O1	0.0633 (15)	0.0806 (17)	0.0476 (13)	−0.0328 (13)	−0.0299 (11)	0.0164 (12)
O2	0.0640 (15)	0.0374 (12)	0.0955 (19)	−0.0198 (11)	−0.0262 (14)	−0.0028 (12)
O3	0.0453 (12)	0.0516 (13)	0.0654 (15)	0.0089 (10)	−0.0076 (11)	−0.0073 (11)
P1	0.0311 (3)	0.0287 (3)	0.0285 (3)	−0.0080 (3)	−0.0054 (3)	0.0009 (3)
Cl1	0.0675 (5)	0.0326 (4)	0.0444 (4)	−0.0180 (3)	0.0051 (3)	−0.0086 (3)
Pd1	0.03329 (16)	0.02494 (15)	0.02737 (15)	−0.00770 (11)	−0.00467 (11)	−0.00023 (10)

### Geometric parameters (Å, °)

**Table:**

C1—C3	1.351 (11)	C24—O2	1.366 (4)
C1—C2	1.375 (12)	C24—C25	1.390 (4)
C1—H1	0.9300	C25—C26	1.380 (4)
C2—C3^i^	1.413 (11)	C25—H25	0.9300
C2—H2	0.9300	C26—H26	0.9300
C3—C2^i^	1.413 (11)	C27—O2	1.436 (4)
C3—H3	0.9300	C27—H27A	0.9600
C11—C16	1.388 (4)	C27—H27B	0.9600
C11—C12	1.395 (4)	C27—H27C	0.9600
C11—P1	1.820 (3)	C31—C32	1.389 (4)
C12—C13	1.387 (4)	C31—C36	1.394 (4)
C12—H12	0.9300	C31—P1	1.815 (3)
C13—C14	1.374 (4)	C32—C33	1.386 (4)
C13—H13	0.9300	C32—H32	0.9300
C14—O1	1.360 (3)	C33—C34	1.393 (4)
C14—C15	1.392 (4)	C33—H33	0.9300
C15—C16	1.382 (4)	C34—O3	1.368 (3)
C15—H15	0.9300	C34—C35	1.381 (4)
C16—H16	0.9300	C35—C36	1.388 (4)
C17—O1	1.441 (4)	C35—H35	0.9300
C17—H17A	0.9600	C36—H36	0.9300
C17—H17B	0.9600	C37—O3	1.434 (4)
C17—H17C	0.9600	C37—H37A	0.9600
C21—C26	1.394 (4)	C37—H37B	0.9600
C21—C22	1.404 (4)	C37—H37C	0.9600
C21—P1	1.821 (3)	P1—Pd1	2.3496 (6)
C22—C23	1.380 (4)	Cl1—Pd1	2.2995 (7)
C22—H22	0.9300	Pd1—Cl1^ii^	2.2995 (7)
C23—C24	1.389 (4)	Pd1—P1^ii^	2.3496 (6)
C23—H23	0.9300		
			
C3—C1—C2	124.8 (9)	C25—C26—C21	121.8 (3)
C3—C1—H1	117.6	C25—C26—H26	119.1
C2—C1—H1	117.6	C21—C26—H26	119.1
C1—C2—C3^i^	118.8 (8)	O2—C27—H27A	109.5
C1—C2—H2	120.6	O2—C27—H27B	109.5
C3^i^—C2—H2	120.6	H27A—C27—H27B	109.5
C1—C3—C2^i^	116.3 (9)	O2—C27—H27C	109.5
C1—C3—H3	121.8	H27A—C27—H27C	109.5
C2^i^—C3—H3	121.8	H27B—C27—H27C	109.5
C16—C11—C12	118.0 (2)	C32—C31—C36	118.4 (2)
C16—C11—P1	121.9 (2)	C32—C31—P1	123.13 (19)
C12—C11—P1	120.1 (2)	C36—C31—P1	118.4 (2)
C13—C12—C11	120.5 (3)	C33—C32—C31	120.5 (3)
C13—C12—H12	119.7	C33—C32—H32	119.8
C11—C12—H12	119.7	C31—C32—H32	119.8
C14—C13—C12	120.7 (3)	C32—C33—C34	120.4 (3)
C14—C13—H13	119.6	C32—C33—H33	119.8
C12—C13—H13	119.6	C34—C33—H33	119.8
O1—C14—C13	116.0 (3)	O3—C34—C35	124.8 (3)
O1—C14—C15	124.5 (3)	O3—C34—C33	115.6 (3)
C13—C14—C15	119.5 (3)	C35—C34—C33	119.7 (3)
C16—C15—C14	119.6 (3)	C34—C35—C36	119.5 (3)
C16—C15—H15	120.2	C34—C35—H35	120.2
C14—C15—H15	120.2	C36—C35—H35	120.2
C15—C16—C11	121.6 (3)	C35—C36—C31	121.4 (3)
C15—C16—H16	119.2	C35—C36—H36	119.3
C11—C16—H16	119.2	C31—C36—H36	119.3
O1—C17—H17A	109.5	O3—C37—H37A	109.5
O1—C17—H17B	109.5	O3—C37—H37B	109.5
H17A—C17—H17B	109.5	H37A—C37—H37B	109.5
O1—C17—H17C	109.5	O3—C37—H37C	109.5
H17A—C17—H17C	109.5	H37A—C37—H37C	109.5
H17B—C17—H17C	109.5	H37B—C37—H37C	109.5
C26—C21—C22	117.5 (2)	C14—O1—C17	118.0 (2)
C26—C21—P1	120.6 (2)	C24—O2—C27	117.4 (3)
C22—C21—P1	122.0 (2)	C34—O3—C37	117.7 (3)
C23—C22—C21	120.9 (3)	C31—P1—C11	106.73 (12)
C23—C22—H22	119.5	C31—P1—C21	104.02 (12)
C21—C22—H22	119.5	C11—P1—C21	104.27 (12)
C22—C23—C24	120.6 (3)	C31—P1—Pd1	111.03 (8)
C22—C23—H23	119.7	C11—P1—Pd1	111.08 (9)
C24—C23—H23	119.7	C21—P1—Pd1	118.81 (9)
O2—C24—C23	115.9 (3)	Cl1^ii^—Pd1—Cl1	180.00 (4)
O2—C24—C25	124.9 (3)	Cl1^ii^—Pd1—P1	88.38 (2)
C23—C24—C25	119.2 (3)	Cl1—Pd1—P1	91.62 (2)
C26—C25—C24	120.0 (3)	Cl1^ii^—Pd1—P1^ii^	91.62 (2)
C26—C25—H25	120.0	Cl1—Pd1—P1^ii^	88.38 (2)
C24—C25—H25	120.0	P1—Pd1—P1^ii^	180.00 (3)
			
C3—C1—C2—C3^i^	−0.6 (15)	P1—C31—C36—C35	176.8 (2)
C2—C1—C3—C2^i^	0.5 (15)	C13—C14—O1—C17	−177.2 (3)
C16—C11—C12—C13	−0.3 (4)	C15—C14—O1—C17	2.6 (5)
P1—C11—C12—C13	−177.6 (2)	C23—C24—O2—C27	176.8 (3)
C11—C12—C13—C14	0.0 (5)	C25—C24—O2—C27	−2.5 (5)
C12—C13—C14—O1	−179.7 (3)	C35—C34—O3—C37	8.5 (5)
C12—C13—C14—C15	0.6 (5)	C33—C34—O3—C37	−171.3 (3)
O1—C14—C15—C16	179.4 (3)	C32—C31—P1—C11	10.3 (3)
C13—C14—C15—C16	−0.9 (5)	C36—C31—P1—C11	−166.3 (2)
C14—C15—C16—C11	0.6 (5)	C32—C31—P1—C21	−99.6 (2)
C12—C11—C16—C15	0.0 (5)	C36—C31—P1—C21	83.8 (2)
P1—C11—C16—C15	177.2 (3)	C32—C31—P1—Pd1	131.5 (2)
C26—C21—C22—C23	−0.3 (4)	C36—C31—P1—Pd1	−45.2 (2)
P1—C21—C22—C23	−179.5 (2)	C16—C11—P1—C31	−80.9 (3)
C21—C22—C23—C24	−0.2 (5)	C12—C11—P1—C31	96.3 (2)
C22—C23—C24—O2	−178.6 (3)	C16—C11—P1—C21	28.8 (3)
C22—C23—C24—C25	0.6 (5)	C12—C11—P1—C21	−154.0 (2)
O2—C24—C25—C26	178.8 (3)	C16—C11—P1—Pd1	157.9 (2)
C23—C24—C25—C26	−0.4 (4)	C12—C11—P1—Pd1	−24.9 (2)
C24—C25—C26—C21	−0.1 (4)	C26—C21—P1—C31	170.1 (2)
C22—C21—C26—C25	0.5 (4)	C22—C21—P1—C31	−10.8 (2)
P1—C21—C26—C25	179.7 (2)	C26—C21—P1—C11	58.4 (2)
C36—C31—C32—C33	1.4 (4)	C22—C21—P1—C11	−122.5 (2)
P1—C31—C32—C33	−175.3 (2)	C26—C21—P1—Pd1	−65.9 (2)
C31—C32—C33—C34	−0.2 (5)	C22—C21—P1—Pd1	113.2 (2)
C32—C33—C34—O3	177.4 (3)	C31—P1—Pd1—Cl1^ii^	−41.94 (9)
C32—C33—C34—C35	−2.4 (5)	C11—P1—Pd1—Cl1^ii^	76.66 (10)
O3—C34—C35—C36	−176.1 (3)	C21—P1—Pd1—Cl1^ii^	−162.45 (10)
C33—C34—C35—C36	3.7 (5)	C31—P1—Pd1—Cl1	138.06 (9)
C34—C35—C36—C31	−2.6 (5)	C11—P1—Pd1—Cl1	−103.34 (10)
C32—C31—C36—C35	0.0 (4)	C21—P1—Pd1—Cl1	17.55 (10)

Symmetry codes: (i) −*x*+1, −*y*+1, −*z*+1; (ii) −*x*, −*y*, −*z*.
